# A study protocol for a predictive model to assess population-based avoidable hospitalization risk: Avoidable Hospitalization Population Risk Prediction Tool (AvHPoRT)

**DOI:** 10.1186/s41512-024-00165-5

**Published:** 2024-02-06

**Authors:** Laura C. Rosella, Mackenzie Hurst, Meghan O’Neill, Lief Pagalan, Lori Diemert, Kathy Kornas, Andy Hong, Stacey Fisher, Douglas G. Manuel

**Affiliations:** 1 Dalla Lana School of Public Health, University of Toronto, 155 College Street, Health Sciences Building 6th Floor, Toronto, ON M5T 3M7 Canada; 2 Institute for Better Health, Trillium Health Partners, Mississauga, ON Canada; 3 Laboratory Medicine and Pathobiology, Temerty Faculty of Medicine, University of Toronto, Toronto, ON Canada; 4 ICES, Toronto, ON M4N 3M5 Canada; 5 PEAK Urban Research Programme, Nuffield Department of Women’s and Reproductive Health, University of Oxford, Oxford, UK; 6 Department of City & Metropolitan Planning, University of Utah, Salt Lake City, UT USA; 7 The George Institute for Global Health, Newtown, NSW Australia; 8 Ottawa Hospital Research Institute, Ottawa, Canada; 9 Statistics Canada, Ottawa, Canada; 10 Department of Family Medicine, University of Ottawa, Ottawa, Canada; 11 School of Epidemiology and Public Health, University of Ottawa, Ottawa, Canada; 12 Bruyère Research Institute, Ottawa, Canada

**Keywords:** Prediction model, Avoidable hospitalization, Ambulatory care sensitive conditions, Study protocol, Population level, Survival analysis

## Abstract

**Introduction:**

Avoidable hospitalizations are considered preventable given effective and timely primary care management and are an important indicator of health system performance. The ability to predict avoidable hospitalizations at the population level represents a significant advantage for health system decision-makers that could facilitate proactive intervention for ambulatory care-sensitive conditions (ACSCs). The aim of this study is to develop and validate the Avoidable Hospitalization Population Risk Tool (AvHPoRT) that will predict the 5-year risk of first avoidable hospitalization for seven ACSCs using self-reported, routinely collected population health survey data.

**Methods and analysis:**

The derivation cohort will consist of respondents to the first 3 cycles (2000/01, 2003/04, 2005/06) of the Canadian Community Health Survey (CCHS) who are 18–74 years of age at survey administration and a hold-out data set will be used for external validation. Outcome information on avoidable hospitalizations for 5 years following the CCHS interview will be assessed through data linkage to the Discharge Abstract Database (1999/2000–2017/2018) for an estimated sample size of 394,600. Candidate predictor variables will include demographic characteristics, socioeconomic status, self-perceived health measures, health behaviors, chronic conditions, and area-based measures. Sex-specific algorithms will be developed using Weibull accelerated failure time survival models. The model will be validated both using split set cross-validation and external temporal validation split using cycles 2000–2006 compared to 2007–2012. We will assess measures of overall predictive performance (Nagelkerke *R*^2^), calibration (calibration plots), and discrimination (Harrell’s concordance statistic). Development of the model will be informed by the Transparent Reporting of a multivariable prediction model for Individual Prognosis or Diagnosis (TRIPOD) statement.

**Ethics and dissemination:**

This study was approved by the University of Toronto Research Ethics Board. The predictive algorithm and findings from this work will be disseminated at scientific meetings and in peer-reviewed publications.

**Supplementary Information:**

The online version contains supplementary material available at 10.1186/s41512-024-00165-5.

## Strengths and limitations

- The Avoidable Hospitalization Population Risk Tool (AvHPoRT) will use routinely collected population-based survey data individually linked to health administrative data in Canada to develop and validate a risk prediction tool for avoidable hospitalizations associated with ambulatory care sensitive conditions
- AvHPoRT will improve existing risk prediction tools for avoidable hospitalization by encompassing non-medical determinants of health such as self-reported demographic characteristics, socioeconomic status, health behaviors, and area-based measures
- Because this model includes non-medical data, we can predict at the population level social determinants of health factors before individuals enter the hospital system, making it useful for public health-focused applications. This addition is a distinct advantage over existing hospital-based algorithms primarily used for triaging people that are already in contact with the acute care system
- The proposed analytic plan follows the recommendations published in the TRIPOD statement for multivariable predictive models to reduce statistical overfitting
- Despite a robust validation approach, including both split set validation and external temporal validation, further validation may be necessary to assess generalizability and calibration for applications outside of Canada
- AvHPoRT can be leveraged by health system decision-makers and planners to identify subgroups of the population at high risk of avoidable hospitalization, to inform population management and prevention approaches, and to estimate the future burden of avoidable hospitalizations in Canada

## Background

Avoidable hospitalizations refer to hospitalizations for conditions that can be prevented, treated, or managed in primary care and, therefore should not necessitate hospitalization [1, 2]. This set of conditions is typically referred to as ambulatory care-sensitive conditions (ACSCs). In the Canadian context, avoidable hospitalizations are defined to include any acute care hospitalization among individuals 0–74 years of age for any of seven ACSCs, including angina, asthma, congestive heart failure, chronic obstructive pulmonary disease, diabetes, epilepsy, and hypertension where the patient is alive at discharge [3]. Variations of this definition exist across health systems, including acute cellulitis, dental conditions, vaccine-preventable conditions (e.g., influenza), and alternative age specifications [4]. Avoidable hospitalizations are an important health system performance indicator that signals poor management of health conditions [5, 6] and inadequate access to quality preventive care in the community [3]. The Canadian Institutes for Health Information (CIHI) estimated that 6.8 million Canadians aged 20–74, have an avoidable hospitalization resulting in approximately 95,000 hospitalizations and 13,000 deaths per year [7, 8]. Despite Canada’s universal healthcare system covering all medically necessary services, social, sex, and geographic inequalities in avoidable hospitalizations exist [7, 9–11]. Additionally, avoidable hospitalizations are expensive for the healthcare system and in 2006, avoidable hospitalizations were estimated to cost the Canadian healthcare system $416 million annually [11].

Several risk factors have been associated with hospitalization for ACSCs, including demographics [12–16], health behaviors [12, 16–18], rurality of residence [6, 13, 19–23], socioeconomic status [9, 10, 16, 24–27], chronic conditions [12, 28, 29], and characteristics related to the organization, structure, and delivery of care [6, 30–33]. Clinical models have been developed to predict the risk of emergency and inpatient hospitalization or re-hospitalization; however, none have specifically been developed for avoidable hospitalizations at the population level and for the Canadian context [34–37]. Canadian researchers have previously developed a simple and complex version of the Hospital Admission Risk Prediction tool to identify those at risk of future 30-day and 15-month all-cause hospitalization using administrative data from Ontario and Manitoba [38] based on hospital utilization variables, achieving moderate discrimination (*c*-statistic 0.66–0.70). Given that survey data were not used, details on socio-demographics (e.g., immigration status, ethnicity), individual-level socioeconomic status (e.g., income, education), social support (e.g., living alone), or health behaviors (e.g., smoking, alcohol consumption, body weight) were not included. Therefore, there are opportunities to improve model performance by adding variables that are lacking in administrative data and creating a model that better informs population health approaches [39]. Furthermore, a model that allows for prediction at the population level contributes by allowing for accurate distribution of risk in the population, which can ensure that strategies for prevention are allocated to the populations that will most likely benefit from attention and outreach. This approach allows resources to be directed in a way that addresses risk along multiple determinants of health and optimizes the impact on populations [40]. This is a critical cornerstone of population health management, an approach increasingly being adopted by many health systems [41].

To address the need for a population-based prediction model for avoidable hospitalizations that encompasses non-medical determinants of health, we propose the development and validation of the Avoidable Hospitalization Population Risk Tool (AvHPoRT). AvHPoRT will use self-reported risk factor information for population-based risk prediction of the first avoidable hospitalization in adults in Canada for seven ACSCs over 5 years: angina, asthma, congestive heart failure, COPD, diabetes, epilepsy, and hypertension. According to recommendations in TRIPOD guidelines, this study protocol pre-specifies the predictor variables and analytic plan for the development and validation of AvHPoRT [42].

## Methods and analysis

### Data sources

#### Canadian Community Health Survey

The Canadian Community Health Survey (CCHS) is a cross-sectional survey that collects information on self-reported sociodemographic characteristics, personal health status, health behaviors, and healthcare utilization for the Canadian population aged 12 years and older [43]. The study base is all Canadian youth and adults, excluding individuals living in certain remote regions of Quebec and Nunavut, full-time members of the Canadian Forces, persons living on reserves and other Aboriginal settlements, and individuals living in institutions [43]. Overall, these exclusions represent < 3% of the Canadian population [43]. Canadian respondents from the following CCHS cycles–1.1 (2000/01), 2.1 (2003/04), 3.1 (2005/06), 2007/08, 2009/10, and 2011/12 will be used to create the overall study cohort and obtain predictor variables. The estimated sample size 394,600.

#### Discharge abstract database and Canadian Vital Statistics Database

The Discharge Abstract Database (DAD) is a national database maintained by the Canadian Institutes of Health Research (CIHI) that contains information on demographic, administrative, and clinical data on hospital inpatient discharges and same-day surgery procedures [44]. All provinces and territories, excluding Quebec, submit information to DAD, representing 75% of all inpatient hospital discharges in Canada. We will use the most recent CCHS-DAD linkage, including data from the fiscal year (FY) 1999/2000–2017/2018. DAD will be used to identify all hospital-based deaths and avoidable hospitalization for seven ACSCs. The Canadian Vital Statistics Database (CVSD) is a national database that includes all deaths registered in Canada, including information on the death date and cause of death. In hospitals, deaths are captured in the DAD. CCHS respondents consented to linkage to health administrative databases, resulting in a linkage rate between the CCHS-DAD and CCHS-CVSD of approximately 85% (excluding Quebec respondents who do not participate) [45, 46]. Further details on the linkage process are described elsewhere [45].

#### Canadian Marginalization Index

The Canadian Marginalization Index (CAN-Marg) is a census-based measure of sociodemographic characteristics, including households and dwellings, material resources, age and labor force, and immigration and visible minority status at the dissemination area level [47]. The dissemination area is the smallest geographic unit for which census information is available, representing approximately 200–700 persons [48]. The 2001, 2006, and 2011 CAN-Marg indices will be used [49]. CAN-Marg is linked to respondents in the CCHS based on their postal code at the time of survey administration.

### Patient and public involvement

We have consulted with partners in public health units across urban and rural Ontario in the development of several population-based risk tools. Our partners at local public health units in Ontario provided feedback on the present protocol, informing the relevant candidate variables and how they would use AvHPoRT once validated. Individual patients will not be involved in the design, conduct, reporting, or dissemination of this research.

### Study design

Using population-based survey data linked to health administrative data, sex-specific AvHPoRT models will be developed and validated using CCHS respondents from the survey years 2000–2012. All analyses will be sex-stratified due to differences in the individual risk factors for first avoidable hospitalizations found in previous studies on this population [16]. As a result, the models will be sex-specific. Two development and validation approaches will be used: split set validation and external validation on a hold-out dataset. Individuals will be followed for 5 years after the CCHS interview date until the first avoidable hospitalization, death, or end of the study period, whichever comes first. For both the development and validation cohorts, respondents will be excluded if they (1) are less than 18 years of age or older than 74 years of age, (2) live in Quebec or the Territories or, (3) are pregnant at the time of the CCHS interview. The lower bound age limitation is due to the differing nature of youth healthcare utilization who are typically under parental or legal guardian care. The upper age limit is due to how CIHI defines avoidable hospitalizations for ACSCs [3]. The rationale is that hospitalizations for the seven conditions captured by definition are deemed less avoidable or completely unavoidable after age 74 due to declines in overall health [3]. Quebec residents will be excluded because this province does not submit DAD records and the Territories will be excluded due to high levels of missing data [45]. Additionally, pregnant respondents will be excluded due to the inability to correctly estimate body mass index (BMI) and the potential for misclassification of baseline covariates (i.e., smoking or alcohol consumption status).

### Identification of potential predictor variables

Predictor variables from the CCHS capture baseline information of the study cohort. We selected variables based on their availability across provinces and cycles, reviewed existing literature on avoidable hospitalization risk factors, observational studies based on survey data linked to avoidable hospitalizations in Canada [16, 27], recommendations from knowledge users in public health, and expertise from our team with prior experience developing and validating predictive algorithms for healthcare use [50], acute and chronic conditions [51–56] and mortality [57, 58]. We limited to variables consistent across provinces and CCHS survey cycles. After screening available predictors, a total of 39 candidate variables were selected, including demographic characteristics (e.g., age, ethnicity), socioeconomic variables (e.g., immigration status, marital status, household income, education), self-perceived measures (e.g., general health, life stress, and community belonging), health behaviors (e.g., cigarette smoking, alcohol consumption, fruit and vegetable consumption, physical activity, BMI), chronic conditions (e.g., diabetes, COPD), healthcare access (e.g., whether respondent has a family physician), use preventive care (ever had a flu shot), and five area-based measures (e.g., four CAN-Marg Indices and one CCHS based individual measure).

### Outcome

Avoidable hospitalization was defined as a hospitalization among adults between 18 and 74 years of age at the time of admission, where discharged alive, and the most responsible diagnosis was one of seven chronic ACSCs: angina (excluding certain cardiac interventions), asthma, congestive heart failure (excluding certain cardiac interventions), chronic obstructive pulmonary disease, diabetes, epilepsy, and hypertension (excluding certain cardiac interventions) (Table 1) [3]. We will consider the first avoidable hospitalization as the outcome. CIHI defines the most responsible diagnosis as the most responsible condition for the patient’s stay in the facility [59]. Per the CIHI definition, certain cardiac interventions performed for hospitalizations due to angina, congestive heart failure, and hypertension are identified using the International Classification of Diseases-9 and the International Classification of Diseases-10 diagnostic codes and will be excluded [3, 60].

**Table 1:** ICD-9 and ICD-10 codes used to capture avoidable hospitalizations as defined by the Canadian Institutes for Health Information

**Condition**	**ICD 9/9-CM and ICD-10-CA**
Grand mal status and other epileptic convulsions	ICD-9/9-CM: 345 ICD-10-CA: G40, G41
Chronic obstructive pulmonary disease	Any most responsible diagnosis code of ICD-9/9-CM: 491, 492, 494, 496 ICD-10-CA: J41, J42, J43, J44, J47 Most responsible diagnosis of acute lower respiratory infection, only when a secondary diagnosis of J44 in ICD-10-CA or 496 in ICD-9/9-CM is also present ICD-9/9-CM: 466, 480–486, 487.0 ICD-10-CA: J10.0, J11.0, J12–J16, J18, J20, J21, J22
Asthma	ICD-9/9-CM: 493 ICD-10-CA: J45
Diabetes	ICD-9: 250.0, 250.1, 250.2, 250.7 ICD-9-CM: 250.0, 250.1, 250.2, 250.8 ICD-10-CA: E10.0, E10.1, E10.63, E10.64, E10.9 E11.0, E11.1, E11.63, E11.64, E11.9 E13.0, E13.1, E13.63, E13.64, E13.9 E14.0, E14.1, E14.63, E14.64, E14.9
Heart failure and pulmonary edema	ICD-9/9-CM: 428, 518.4 ICD-10-CA: I50, J81
Hypertension	ICD-9/9-CM: 401.0, 401.9, 402.0, 402.1, 402.9 ICD-10-CA: I10.0, I10.1, I11
Angina	ICD-9: 411, 413 ICD-9-CM: 411.1, 411.8, 413 ICD-10-CA: I20, I23.82, I24.0, I24.8, I24.9
Cardiac procedure codes for exclusion	Codes for exclusion applied to heart failure and pulmonary edema, hypertension, and angina: CCP: 47^^, 480^–483^, 489.1, 489.9, 492^–495^, 497^, 498^ ICD-9-CM: 336, 35^^, 36^^, 373^, 375^, 377^, 378^, 379.4–379.8 CCI: 1.HA.58.^^, 1.HA.80.^^, 1.HA.87.^^, 1.HB.53.^^, 1.HB.54.^^, 1.HB.55.^^, 1.HB.87.^^, 1.HD.53.^^, 1.HD.54.^^, 1.HD.55.^^, 1.HH.59.^^, 1.HH.71.^^, 1.HJ.76.^^, 1.HJ.82.^^, 1.HM.57.^^, 1.HM.78.^^, 1.HM.80.^^, 1.HN.71.^^, 1.HN.80.^^, 1.HN.87.^^, 1.HP.76.^^, 1.HP.78.^^, 1.HP.80.^^, 1.HP.82.^^, 1.HP.83.^^, 1.HP.87.^^, 1.HR.71.^^, 1.HR.80.^^, 1.HR.84.^^, 1.HR.87.^^, 1.HS.80.^^, 1.HS.90.^^, 1.HT.80.^^, 1.HT.89.^^, 1.HT.90.^^, 1.HU.80.^^, 1.HU.90.^^, 1.HV.80.^^, 1.HV.90.^^, 1.HW.78.^^, 1.HW.79.^^, 1.HX.71.^^, 1.HX.78.^^, 1.HX.79.^^, 1.HX.80.^^, 1.HX.83.^^, 1.HX.86.^^, 1.HX.87.^^, 1.HY.85.^^, 1.HZ.53 rubric (except 1.HZ.53.LA-KP), 1.HZ.54.^^, 1.HZ.55 rubric (except 1.HZ.55.LA-KP), 1.HZ.56.^^, 1.HZ.57.^^, 1.HZ.59.^^, 1.HZ.80.^^, 1.HZ.85.^^, 1.HZ.87.^^, 1.IF.83.^^, 1.IJ.50.^^, 1.IJ.54.GQ-AZ, 1.IJ.55.^^, 1.IJ.57.^^, 1.IJ.76.^^, 1.IJ.80.^^, 1.IJ.86.^^, 1.IK.50.^^, 1.IK.57.^^, 1.IK.80.^^, 1.IK.87.^^, 1.IN.84.^^, 1.LA.84.^^, 1.LC.84.^^, 1.LD.84.^^, 1.YY.54.LA-NJ, 1.YY.54.LA-FS, 1.YY.54.LA-NM, 1.YY.54.LA-FR, 1.YY.54.LA-FU

^ : Any single additional character

^^ : Any two additional characters

### Sample size

To increase the likelihood of developing a robust prediction model, we will combine multiple cycles of the CCHS to increase the sample size. In taking this approach, we aim to minimize the potential for model overfitting and to increase the precision of predictions. A recent Canadian study by our team examined individual and area-level factors that were associated with the risk of avoidable hospitalizations, which included the first 6 cycles of the CCHS linked to the DAD with study exclusions that are consistent with those proposed in this protocol [27]. Therefore, we anticipate approximately 389,100 respondents with 8,500 (2.2%) avoidable hospitalizations [16]. Following CCHS sampling methods [60], we anticipate an approximately equal number of males and females, resulting in approximately 195,000 observations for each sex-specific model.

We calculated the minimum sample size for AvHPoRT using the *pmsampsize* package in R according to the approach proposed by Riley et al., which considers context-specific factors including the total number of study participants, the proportion of the outcome in the study population, and the anticipated predictive performance of the model [61, 62]. Assuming a Nagelkerke’s R of 16.5% based on a comparable risk prediction tool for Ontario [38], with an outcome proportion of 2.1%, 163 parameters (82 from all variables in Table 2 plus 81 for testing interactions with linear age), and a shrinkage factor of 0.90 the minimum sample size is 16,664 respondents with 333 events per model. The expected sample size surpasses these minimum estimates, and as a result, we anticipate a sufficient sample for our proposed analyses.

**Table 2:** Pre-specification of predictor variables for AvHPoRT with initial degrees of freedom (df)

**Variable grouping**	**Variable**	**Definition**	**df**	**Scale**
**Demographics**	**Age** (years)	5-knot restricted cubic spline	4	Continuous
	**Self-identified ethnicity**		1	Dichotomous
	White			
	Non-white			
**Socioeconomic status and self-perceived measures**	**Immigration status**		2	Categorical
	Canadian born			
	Recent immigrant	Immigrated < 10 years		
	Non-recent immigrant	Immigrated ≥ 10 years		
	**Marital status**		2	Categorical
	Single never married			
	Domestic partner (married/common law)			
	Widowed/separated/divorced			
	**Household income**		4	Ordinal
	Quintile 1	Lowest 20%		
	Quintile 2			
	Quintile 3			
	Quintile 4			
	Quintile 5	Highest 20%		
	**Education**		3	Ordinal
	Less than secondary school graduation			
	Secondary school graduation			
	Some post-secondary education			
	Post-secondary completed			
	**Self-perceived general health**		4	Ordinal
	Excellent			
	Very good			
	Good			
	Fair			
	Poor			
	**Self-perceived life stress**		4	Ordinal
	Not at all			
	Not very			
	A bit stressful			
	Quite a bit			
	Extremely stressful			
	**Self-perceived community belonging**		3	Ordinal
	Very strong			
	Somewhat strong			
	Somewhat weak			
	Very weak			
**Health behaviors**	**Cigarette smoking**		4	Categorical
	Non-smoker	Never a smoker or former occasional smoker with < 100-lifetime cigarettes		
	Former heavy smoker	Former smoker [≥ 1 pack (25 cigarettes)/day]		
	Former light smoker	Former smoker [< 1 pack (25 cigarettes)/day]		
	Heavy smoker	Current smoker [≥ 1 pack (25 cigarettes)/day]		
	Light smoker	Current smoker [< 1 pack (25 cigarettes)/day]		
	**Alcohol consumption**		3	Categorical
	Non-drinker	No alcohol consumption in the last 12 months or drink frequency fewer than once a week		
	Light drinker	Alcohol consumption frequency at least once a week and 0–2 (females) or 0–3 (males) drinks in the previous week		
	Moderate drinker	3–14 (females) or 4–21 (males) drinks in the previous week		
	Heavy drinker	≥ 14 (females) or ≥ 21 (males) drinks in the previous week, or binging behavior on a weekly basis (≥ 5 drinks on any occasion)		
	**Daily fruit and vegetable consumption**		2	Ordinal
	Low consumption	0 to less than 3 times daily		
	Medium consumption	3 to less than 6 times daily		
	High consumption	6 or more times daily		
	**Leisure physical activity** (kcal/kg/day)		2	Ordinal
	Active	3.0 or more metabolic equivalents per day		
	Moderate	1.5–2.9 metabolic equivalents per day		
	Inactive	Less than 1.5 metabolic equivalents per day		
	**Body mass index** (BMI) (kg/m^2^)	5-knot restricted cubic spline	4	Continuous
**Chronic conditions**	**Self-reported chronic conditions diagnosed by a health professional**		17	Dichotomous
	Including asthma, arthritis, back problems, high blood pressure, migraines, emphysema, chronic obstructive pulmonary disease, diabetes, heart disease, cancer, intestinal ulcers, stroke, urinary incontinence, bowel disease, mood disorder, or anxiety disorder	Yes/no for each individual chronic condition		
**Healthcare utilization**	Ever had a flu shot	Yes/no	1	Binary
	Has a regular medical doctor	Yes/no	1	Binary
**Area-based variables**	**Rurality**		1	Dichotomous
	Population center	Population of at least 1000 and a density of ≥ 400 people per square kilometer based on current census population counts		
	Rural area	Population concentration or densities below the urban threshold based on current census population counts		
	**Material deprivation**		4	Ordinal
	Quintile 1	Least deprived		
	Quintile 2			
	Quintile 3			
	Quintile 4			
	Quintile 5	Most deprived		
	**Ethnic diversity**		4	Ordinal
	Quintile 1	Least diverse		
	Quintile 2			
	Quintile 3			
	Quintile 4			
	Quintile 5	Most diverse		
	**Residential instability**		4	Ordinal
	Quintile 1	Least unstable		
	Quintile 2			
	Quintile 3			
	Quintile 4			
	Quintile 5	Most unstable		
	**Dependency**		4	Ordinal
	Quintile 1	Least dependent		
	Quintile 2			
	Quintile 3			
	Quintile 4			
	Quintile 5	Most dependent		
	**Area-level household income**		4	Ordinal
	Quintile 1	Lowest		
	Quintile 2			
	Quintile 3			
	Quintile 4			
	Quintile 5	Highest		

### Analysis plan

The analytic plan was developed following the guidelines for prediction models by Steyerberg [63] and Harrell [64]. This plan was constructed after accessing our study cohort but before model fitting or evaluation of descriptive statistics examining relationships between predictor variables and the outcome. Important considerations that informed the analytic approach include full pre-specification of predictor variables and implementation of flexible functions for continuous variables. As a final step, we will verify the sequential addition of predictors using the least absolute shrinkage and selection operator (LASSO) [65]. In the case of categorical variables, if the LASSO selects only one of the values in a categorical variable, we will manually test after to see if keeping the variable in the model or removing it will give better model performance. In agreement with recent publications that have called for improvements to the design and reporting of prediction models [66], we have proposed this study protocol to help improve the transparency of the model-building process, increase the robustness of our prediction model, and limit type I errors. Data cleaning and coding of predictors will be conducted in SAS V.9.4, and model development and validation will be carried out in R using Harrell’s *HMisc* [67] and *rms* packages of functions, *‘survey’* among others [68]. This protocol was developed following recommendations of the TRIPOD statement for multivariable predictive models, which will also inform the reporting of AvHPoRT [42, 69].

### Coding and cleaning of predictor variables

All data cleaning and coding of predictor variables will occur prior to examining exposure-outcome relationships. Descriptive statistics and boxplots will be used to examine the width of distributions. Continuous variables with highly skewed distributions will be winsorized to the 99.5th percentile, which will set all extreme values or outliers to the 99.5th percentile. We will also take into account how predictor variables have been modeled in prior risk prediction models [50–55, 57]. Consistent with prior model development, some predictor variables will be derived based on a combination of variables in the CCHS. For example, alcohol consumption will be defined based on a combination of three variables, including whether a respondent reported drinking in the past year, the number of times the respondent drank in the past week, and the total number of drinks consumed in the past week to create a final variable with four categories. A BMI correction equation will be used to reduce the bias in self-reported height and weight [70]. All predictor variables and their definitions have been pre-specified to minimize the possibility of overfitting (Table 2). Additional details on the CCHS questions used to create health risk behavior variables are available as a supplementary file (see online Supplementary file 1).

### Approach to missing data

Given the limitations associated with complete case analysis, including inefficiency and selection bias, multiple imputations will be used to assign values to missing predictor variables using the *mice* package in R, which imputes incomplete multivariate data by chained equations (mice) [71, 72]. Using the mice procedures [73], we will incorporate the full list of predictors, the outcome, and auxiliary variables (i.e., variables that are not predictors but may be useful in lending information to impute missing values) in the imputation procedure. A total of up to five imputed datasets will be generated. The final model will be run on each imputed dataset separately, and the results will be combined using the rules recommended by Rubin and Schenker [72] to account for imputation uncertainty. Missing rates in the data source are known to be rather low (< 5%) and hence no attempts are made to check sensitivity to the MAR assumption. The assessment of model performance based on multiple imputations is a challenging task and we will closely follow the guidance provided by Wood et al. [74].

### Model specification

Sex-specific models will be developed using the pre-specified predictor variables outlined in Table 2. Continuous predictors will be modeled flexibly using restricted cubic splines with piecewise cubic functions smoothed at the knot placements based on Harrell’s percentile recommendations [64]. Table 2 presents the initial model specification which includes 82 degrees of freedom. During the model-building process, we will also examine alternate variable forms used in prior models to perform best. For example, we aim to include BMI as both a continuous predictor (i.e., body mass index as specified in Table 2); however, will also test BMI in its ordinal form using the World Health Organization classifications for BMI (underweight, normal weight, overweight, obese type I, obese type II, and obese type III). We will compare the continuous form with the categorical form using measures of overall predictive performance, model fit, discrimination, calibration, and information criterion (e.g., AIC and BIC). The form of the predictor that improves the overall model fit will be chosen for the final model. Variables will be centered on their means for ease of re-calibration in new populations, which can center data on local means. In addition, we will consider interaction terms with linear age and all other variables listed in Table 2.

Initially, we will fit a full multivariable model containing all prior predictors as specified in Table 2. Then as a subsequent step, we will apply the least absolute shrinkage and selection operator (LASSO) will be used for variable selection [75]. Since the value of lambda plays a very important role for LASSO, a k-fold cross-validation method will be used on the derivation cohort to select the appropriate value of lambda by comparing the partial-likelihood deviance [74]. Should predictors be selected in one imputed dataset and not the other, careful consideration will be made to decide which predictors will be chosen for the final model. For example, to build a more comprehensive model, both predictors may be kept; however, if both predictors are from the same casual path, then we may choose only one so they do not interfere with each other and reduce the model performance. Each predictor will be carefully considered in this way and documented in the final paper for AvHPoRT.

### Model estimation

The 5-year risk of having an avoidable hospitalization will be assessed using sex-specific Weibull accelerated failure time survival models. Our team’s previous work using development and validation methods has demonstrated that Weibull models perform well for population-based prediction models [49–54]. Using a survival model will also properly handle premature mortality, which is the competing risk of avoidable hospitalizations, by censoring individuals once they have had a premature mortality.

To confirm the parametric assumptions of the Weibull model are met, stratified Kaplan-Meier estimates will be made and log-log plots will be plotted against log survival time to confirm they are approximately linear and parallel [62]. The proportional hazards assumption will be checked by plotting stratified log cumulative hazards and assessing the Schoenfeld residuals. Predictor-time interaction terms will be added to the model if required. To ensure the proposed analysis is representative of the Canadian population, survey weights provided by Statistics Canada will be used to account for complexities in the CCHS survey design.

### Model validation

The model will first be derived using the first three CCHS cycles ((1.1 (2000/01), 2.1 (2003/04), 3.1 (2005/06)). We will internally validate using a split sample approach where the 70% development model will be applied to the remaining 30%. The model will then be externally validated in a hold-out dataset using all data of the last three CCHS cycles (2007/08, 2009/10, and 2011/12). Once the final model is determined, all data will be combined to estimate the final application of the model. In order to assess if the model performs similarly across age, income quintile, region, sex, immigration, and education, we will examine performance across geography, levels of education, income, and immigration status.

### Assessment of model performance

The overall predictive performance in the derivation, validation, and combined cohorts will be examined and reported according to overall measures of predictive accuracy, discrimination, and calibration. Measures of overall predictive accuracy include the proportion of variance explained by predictive variables (i.e., Nagelkerke’s *R*^2^) and the Integrated Brier score [76]. Discrimination is defined as the ability of a model to correctly differentiate between respondents who develop the outcome vs respondents who do not [76]. Discrimination will be evaluated using Harrell’s concordance statistic with 95% confidence intervals estimated using bootstrap samples. In the evaluation of predictive performance, Steyerberg [63] and Cook [77, 78] recommend routine assessment of calibration and calibration slopes. Model calibration will primarily be evaluated by visually comparing the observed and predicted risk of avoidable hospitalization over deciles of predicted risk using calibration points over different periods of time (e.g., 1, 3, and 5 years). We will prioritize visual inspection of calibration plots which is less influenced by a large sample size, in contrast to formal statistical significance testing [79]. Calibration slopes will be created by regressing the outcome in the validation cohort on the predicted risk of avoidable hospitalization, thereby reflecting true differences in the effects of predictor variables and the effect of overfitting to the development cohort. Perfect calibration is indicated by a slope of 1 which will be evaluated using the Wald or likelihood ratio tests. Adequate calibration across subgroups defined above will be defined as a relative difference of less than 20% between observed and predicted risk within subgroups. The distribution of the risk of avoidable hospitalization will be assessed for extreme values and outliers and clearly reported in our final paper.

### Model presentation

The final AvHPoRT model will be presented with both beta coefficients as well as hazard ratios and corresponding 95% confidence intervals. With population-based predictions as the primary goal, the model presentation will also include the model coefficients. In addition, a figure of 5-year risk across all individuals will be generated to describe the distribution of risk of avoidable hospitalization. The planning and dissemination of our model is informed by the Population Health Planning Knowledge-to-Action Model [80] developed and evaluated by our team [81]. Once the model is validated, we will carry out training workshops to build health system capacity in a local context where the model is being used. Specifically, we will develop training workshops that our team holds with local public health units to demonstrate how risk prediction tools can be leveraged to inform decision-making and planning in their setting. To increase the accessibility of AvHPoRT, the program to run the model will be made available in several statistical packages and formats, including user-friendly, point-and-click web applications. It is important to note that the use of this model to plan interventions should be accompanied by a careful evaluation of the benefit achieved, preferably accompanied by high-quality evidence on the efficacy of the proposed interventions. Furthermore, the variables that are used in the risk assessment may also be known to healthcare professionals taking care of patients or the general population. Individuals, health care providers, and system planners may act on the information from the variables in the models in different ways all contributing to outcomes in the population.

## Discussion

Avoidable hospitalizations are an important health system indicator that is meaningful in the context of health system evaluation and improvement. Existing risk prediction tools for avoidable hospitalizations and other similar endpoints (i.e., emergency, inpatient, and re-admission) have not been developed using population survey data and do not contain important modifiable risk factors such as socioeconomic status and health behaviors. Importantly, existing tools rely on data from individuals once they have already entered the acute care system, which does not support public health prevention approaches that are often led by public health outside the acute care sector. Currently, health decision-makers across the health system do not have a simple and streamlined approach to estimate the incidence of avoidable hospitalization tailored to their local populations, which can further complement efforts at the individual level. The ability to accurately predict the incidence of avoidable hospitalizations at the population level using modifiable risk factors will inform both broad and targeted prevention approaches and support population health management. The purpose of our model is to estimate the risk of the first avoidable hospitalization within a 5-year period as that is the indicator defined in the Canadian context where the model will be used. Future models can also consider subsequent avoidable hospitalizations using survival models that consider multiple events. This is a future application not covered in this analysis.

## Limitations

There are some limitations to the proposed development and validation of AvHPoRT. First, the study population will be limited to CCHS respondents who agreed to share and link their responses (> 80% of respondents) with the DAD. To accommodate for these small underlying differences between the subset of respondents who agreed to share their responses and the full CCHS cohort, we will use survey weights provided by Statistics Canada [82]. Additionally, the data that will be used to develop AvHPoRT is based on self-reported predictors captured at a single point in time with the potential for systematic and non-directional misclassification error. Despite this, variables from self-reported CCHS data have produced robust prediction models in the past [50–55, 57]. Furthermore, the main reason to use such data is that it is regularly and widely available to health planners. While we anticipate that AvHPoRT will be representative of the majority of the Canadian adult population (98%), some groups are not captured in the CCHS sampling frame, including on-reserve Indigenous peoples. This is an important consideration because persons of Indigenous identity have been reported to have higher rates of avoidable hospitalizations in Canada [83]. Due to limits in data sharing and availability, residents of Quebec and the Territories will also be excluded and thus the model will not necessarily apply in those regions. The use of a risk model as a tool to plan interventions requires further considerations, including the need for high-quality evidence that demonstrates the efficacy of proposed interventions. The generation of high-quality evidence on interventions is needed to achieve beneficial population outcomes informed by the tool. In addition, despite effectively identifying high-risk groups with these tools, it is important to note that accessibility may be a factor preventing groups from benefiting from policies, programs, or interventions. Therefore, in addition to the availability of high-quality evidence on interventions, accessibility is an additional factor that must be considered. Finally, users must assess the impact of potential extreme and rare values on subgroups of risk to ensure they are not overly influential or creating instability.

## Conclusions

We anticipate that AvHPoRT will be a valuable addition to the tools used by regional, provincial, and national decision-makers to support ongoing population health management and public health planning.

### Supplementary Information

**Additional file 1.** Summary of health risk behaviour questions and response options from the Canadian Community Health Survey.

## Data Availability

The data used to generate the study cohort are available only through one of the Statistics Canada Research Data Centres, but restrictions apply to the availability of these data, which were used under license for the current study, and so are not publicly available. Data are however available from the authors upon reasonable request and with permission of the Research Data Centre.

## Abbreviations

ACSC: Ambulatory care sensitive condition
CIHI: Canadian Institutes for Health Information
CCHS: Canadian Community Health Survey
DAD: Discharge Abstract Database
BMI: Body mass index
TRIPOD: Transparent reporting of a multivariable prediction model for individual prognosis or diagnosis
Can-MARG: Canadian Marginalization Index
df: Degrees of freedom
AIC: Akaike information criterion
BIC: Bayesian information criterion
