# Supplementary material for: A study protocol for a predictive model to assess population-based avoidable hospitalization risk: Avoidable Hospitalization Population Risk Prediction Tool (AvHPoRT)
Source: Diagn Progn Res. 2024 Feb 6;8:2. doi: 10.1186/s41512-024-00165-5 (PMC10845544; doi:10.1186/s41512-024-00165-5)
Supplement: Supplementary file 1 — Additional file 1. Summary of health risk behaviour questions and response options from the Canadian Community Health Survey. [file 41512_2024_165_MOESM1_ESM.docx]

**Additional File 1**. Summary of health risk behaviour questions and response options from the Canadian Community Health Survey

| **Health Risk Behaviours** | **Question** | **Response** |
| --- | --- | --- |
| **Leisure physical activity** | Have you done any of the following in the past 3 months, that is, from [date three months ago] to yesterday? | No leisure physical activity or engaged in physical activity (1-99 times) including activities such as Walking for exercise, Gardening or yard work, Swimming, Bicycling, Popular or social dance, Home exercises, Ice hockey, Ice skating, In-line skating or rollerblading, Jogging or running, Golfing, Exercise class or aerobics, Downhill skiing or snowboarding, Bowling, Baseball or softball, Tennis, Weight-training, Fishing, Volleyball, Basketball, Soccer, Any other activity |
|  | About how much time did you spend on each occasion? | 1 to 15 minutes, 16 to 30 minutes, 31 to 60 minutes, More than one hour |
| **Cigarette smoking** | In your lifetime, have you smoked a total of 100 or more cigarettes (about 4 packs)? | Yes, No |
|  | Have you ever smoked a whole cigarette? | Yes, No |
|  | At the present time, do you smoke cigarettes daily, occasionally or not at all? | Daily, Occasionally, Not at all |
|  | How many cigarettes do you smoke each day now? | 1 to 99 |
|  | On the days that you do smoke, how many cigarettes do you usually smoke? | 1 to 41 |
|  | In the past month, on how many days have you smoked 1 or more cigarettes? | 0 to 30 |
|  | Have you ever smoked cigarettes daily? | Yes, No |
|  | When did you stop smoking? | Less than one year ago?, 1 year to less than 2 years ago?, 2 years to less than 3 years ago?, 3 or more years ago? |
|  | How many cigarettes did you usually smoke each day? | 1 to 99 |
|  | When did you stop smoking daily? | Less than one year ago?, 1 year to less than 2 years ago?, 2 years to less than 3 years ago?, 3 or more years ago? |
|  | Was that when you completely quit smoking? | Yes, No |
|  | When did you stop smoking completely? Was it: | Less than one year ago?, 1 year to less than 2 years ago?, 2 years to less than 3 years ago?, 3 or more years ago? |
| **Daily fruit and vegetable consumption** | How often do you usually drink fruit juices such as orange, grapefruit or tomato? | 0 to 99 [per day/per week/per month/per year], Never |
|  | Not counting juice, how often do you usually eat fruit? | 0 to 99 [per day/per week/per month/per year], Never |
|  | How often do you (usually) eat green salad? | 0 to 99 [per day/per week/per month/per year], Never |
|  | How often do you usually eat potatoes, not including French fries, fried potatoes, or potato chips? | 0 to 99 [per day/per week/per month/per year], Never |
|  | How often do you (usually) eat carrots? | 0 to 99 [per day/per week/per month/per year], Never |
|  | Not counting carrots, potatoes, or salad, how many servings of other vegetables do you usually eat? | 0 to 99 [per day/per week/per month/per year], Never |
| **Alcohol consumption** | During the past 12 months, have you had a drink of beer, wine, liquor or any other alcoholic beverage? | Yes, No |
|  | During the past 12 months, how often did you drink alcoholic beverages? | Less than once a month, Once a month, 2 to 3 times a month, Once a week, 2 to 3 times a week, 4 to 6 times a week, Every day |
|  | How often in the past 12 months have you had 5 or more drinks on one occasion? | Never, Less than once a month, Once a month, 2 to 3 times a month, Once a week, More than once a week |
|  | Thinking back over the past week, that is, from [date last week] to yesterday, did you have a drink of beer, wine, liquor or any other alcoholic beverage? | Yes, No |
|  | Starting with yesterday, that is [insert day], how many drinks did you have? [Sunday/Monday/Tuesday/Wednesday/Thursday/Friday/Saturday]? | 0 to 99 |
